# Early discontinuation of immune checkpoint inhibitor therapy prior to disease progression in patients with metastatic non-small cell lung cancer: a survival analysis

**DOI:** 10.3389/fonc.2024.1417175

**Published:** 2024-06-21

**Authors:** Blake J. McKinley, Tanmayi S. Pai, Emily B. Wolf, Shenduo Li, Guilherme Sacchi de Camargo Correia, Yujie Zhao, Rami Manochakian, Yanyan Lou

**Affiliations:** ^1^ Department of Internal Medicine, Mayo Clinic, Jacksonville, FL, United States; ^2^ Division of Hematology and Medical Oncology, Department of Internal Medicine, Mayo Clinic, Jacksonville, FL, United States

**Keywords:** NSCLC, early discontinuation, progression-free survival, overall survival, stage IV, metastatic, immunotherapy, immune checkpoint inhibitor

## Abstract

**Introduction:**

Limited survival data are available for patients with metastatic non-small cell lung cancer (mNSCLC) who stop immune checkpoint inhibitor therapy (ICI) early for reasons other than progression of disease (POD), such as immune-related adverse events (irAEs).

**Methods:**

We conducted a retrospective observational study of all patients with mNSCLC treated with ICIs, with or without combination chemotherapy, at 3 Mayo Clinic sites between 2011 and 2022. Separate analyses were conducted at 6- and 12-month intervals. Patients who discontinued ICI due to POD prior to these time points were excluded from the analysis.

**Results:**

A total of 246 patients with stage IV NSCLC used ICIs. Patients were then excluded if they had experienced POD prior to 6 or 12 months, resulting in 81 and 63 patients, respectively, for each timepoint. Sixty-four patients continued treatment beyond 6 months and were found to have longer progression-free survival (PFS) compared to the 17 patients who discontinued treatment (22.8 months vs 11.8 months, *P* =1.1E-04), as well as a significant increase in overall survival (OS) (33.9 months vs 14.4 months, *P* =7.2E-08). Forty patients continued treatment beyond 12 months and had longer PFS compared to the 23 patients that discontinued treatment (27.9 months vs 14.8 months, *P* =1.1E-04), as well as a significant increase in OS (39.7 months vs 18.0 months, *P* =2.0E-07). The most common reason for ICI discontinuation was irAEs. Other common reasons for stopping ICI were non-irAEs and stable disease. At both time points, 12 patients continued or restarted ICI after experiencing an irAE, and 2 patients experienced recurrent/new grade 1–2 irAEs. More patients continued/rechallenged with ICI after experiencing an irAE in the groups that continued ICI compared to those that discontinued ICI.

**Conclusions:**

Patients with mNSCLC and no POD who continued ICI beyond 6 months and 12 months, experienced significantly increased PFS and OS compared to patients who discontinued ICI, with larger increases in those who continued ICI past 12 months. Oncology providers should discuss the survival benefits of continuing ICI and offer support to overcome obstacles to continuation of treatment, if possible, particularly management of grade 1 and 2 irAEs.

## 1 Introduction

Lung cancer is the leading cause of cancer death worldwide (1). Non‐small cell lung cancer (NSCLC) accounts for 85%–90% of all lung cancers (2). Lung cancer is frequently identified in advanced stages. Immune checkpoint inhibitor therapies (ICI) that target the PD‐1/PD‐L1 axis have become first-line treatment options as monotherapy or in combination with chemotherapy for patients with stage IV NSCLC and shown significantly improved clinical outcomes in some patients (3). Currently, multiple ICIs are FDA approved for treatment of NSCLC. Pembrolizumab was approved in 2016 by the Food and Drug Administration (FDA) as first-line monotherapy in treatment‐naive metastatic NSCLC with a PD‐L1 tumor proportion score (TPS) ≥ 50% (4). In 2019, the FDA expanded pembrolizumab to be used as first-line treatment in metastatic NSCLC for patients with PD‐L1 TPS ≥ 1% and no EGFR or ALK genomic aberrations (5). Atezolizumab, nivolumab, as well as other ICIs have also been shown to offer survival benefits in the first-line setting as monotherapy or combination therapies in metastatic NSCLC (6).

Based on the trials that led to the approval of ICIs, the National Comprehensive Cancer Network guidelines (NCCN) suggest that patients with metastatic NSCLC receive maintenance immunotherapy for 2 years if they receive front-line ICI (7). Discontinuation of ICI treatment after two years in patients with a complete or partial response is recommended for most agents, but not yet universally practiced (8). Recent studies have investigated if there is any benefit to continuation of ICI past 2 years and have found no statistical survival difference in patients who continue vs discontinue ICI (9, 10). However, most patients do not complete 2 years of ICI. One study of 756 patients who received nivolumab or pembrolizumab revealed that only 12% completed 2 years of therapy (10). The most common reason for ICI discontinuation is progression of disease (POD). Some patients discontinue therapy for other reasons, including immune-related adverse events (irAEs), other adverse events, patient preference, cost, etc. Our study aims to understand the differences in outcomes in patients who stop ICI prior to 2 years for reasons other than POD, and to determine if patients with no POD at 6 months and 12 months after ICI initiation experience a survival benefit if they continue ICI. Clinically, this information would be useful for physicians to better inform patients of survival outcomes if ICI is continued beyond these time points.

## 2 Materials and methods

### 2.1 Patients and data analysis

We conducted a retrospective study of all patients seen at our institution from 2011 to 2022 who had tissue confirmation of metastatic NSCLC and were subsequently treated with ICI. Stage IV disease was defined by AJCC 8^th^ edition. All histologic subtypes of NSCLC were included. All types of ICI were included. Patients previously treated with local therapies, such as surgery or radiation therapy, or who had received systemic therapy prior to ICI were included. Patients who received concurrent chemotherapy with ICI were also included. Patients were excluded if they received dual ICI with CTLA-4 inhibitors. Patients were excluded if they had received ICI in stage III disease prior to progressing to stage IV: patients with history of stage III unresectable NSCLC and received concurrent chemotherapy and radiation followed by maintenance durvalumab (11, 12). Overall survival was defined as time from ICI start date until death or time of last contact with the patient. Progression-free survival was defined as the time from the ICI start date to POD. POD was determined to be the first documented event of tumor progression by evidence on imaging per RECIST criteria and confirmed by the treating physician. If no POD was identified prior to the patient’s death, then death was also considered POD.

**Figure 1:**
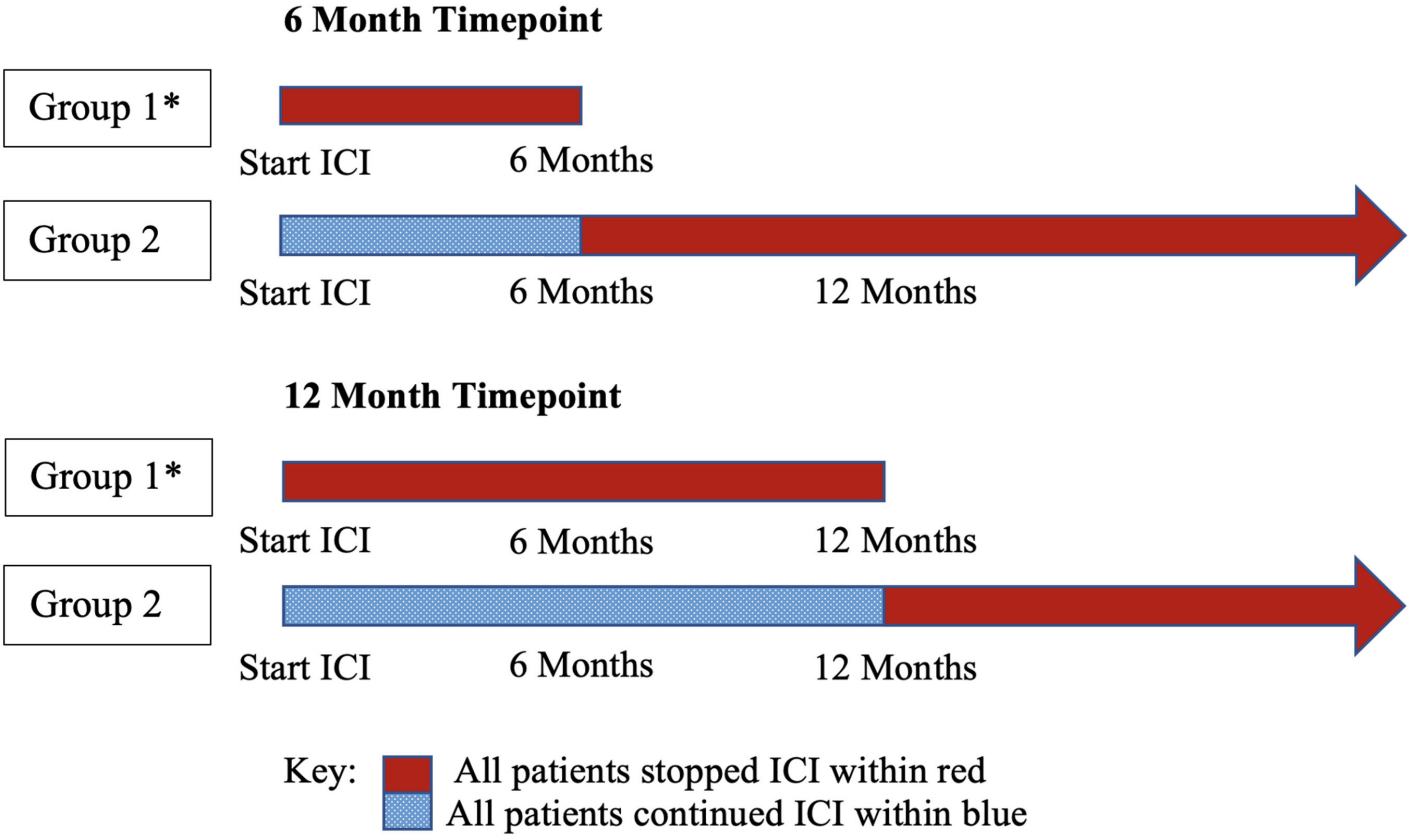
Group comparisons by timepoint: two independent analyses. Group 1 was compared to group 2 to determine survival outcome differences. * All patients in group 1 stopped ICI without POD. ICI, immune checkpoint inhibitor therapy.

Data was obtained from the Mayo Clinic electronic medical record. 1300 NSCLC patient charts from 2011 to 2022 were reviewed. Two investigators performed a quality review. Outcomes were investigated by comparing groups of patients with no POD who stopped ICI prior to 6 or 12 months and those who continued ICI past 6 or 12 months (**Figure 1**). Patients who were included in the 6-month and 12-month cohorts had no POD and stopped ICI before 7 months and 13 months, respectively, to account for differences in timing of ICI cycles that may have extended a few days after the 6- or 12-month time points. Patients who stopped ICI within a two-month period between the last ICI dose and the time of documented POD were considered to have stopped ICI due to POD, not other reasons. This timeframe helped to clearly delineate between patients who stopped ICI due to POD and those who stopped for other reasons, including the time between the last ICI dose and restaging scans, which is when POD could be discovered and ICI could be stopped. This study was approved by the institutional review board at Mayo Clinic Florida.

### 2.2 Statistical analysis

Statistical analyses were performed for each cohort by conducting f-test, two-sample for variances to determine if the comparison groups had statistically different variances. A two-sample t-test was used, assuming unequal or equal variances based on the f-test. A chi-square test of independence was performed to compare if there was a significant difference in irAE incidence, performance status, and PD-L1 expression between groups. A p-value of 0.05 was used to determine statistical significance.

## 3 Results

### 3.1 Patient characteristics

A total of 1300 patient charts were reviewed; 353 patients used ICI, and 246 had stage IV disease. Using this data set, independent analyses were performed at timepoints 6 months and 12 months (**Figure 1**). For each timepoint, patients were excluded if they experienced POD prior to 6 or 12 months.

#### 3.1.1 Six-month timepoint analysis cohort

In the 6-month analysis of 246 patients with stage IV disease, 165 patients were excluded because they experienced POD prior to 6 months, resulting in 81 total patients: 17 patients without POD discontinued ICI prior to 6 months and were compared to 64 patients that continued ICI beyond 6 months. The 81 patients had a median age of 67 (range, 46 - 94); 85.2% were white, 53.1% were male, and 81.5% had a history of tobacco use. Adenocarcinoma (80.2%) was the most common histologic type, followed by squamous cell carcinoma (11.1%). The most common site of metastasis was bone (24.2%), followed by CNS (16.8%), lymph nodes (15.8%), contralateral lung (12.6%), and pleura (including pleural effusion, 12.6%). 75.3% of patients had stage IV disease at the initial diagnosis. 60.5% of patients had received ICI as their first-line systemic therapy, and 29.6% received it as their second. Pembrolizumab was the most frequently used ICI (84.8%), followed by atezolizumab (12.6%) and nivolumab. 43.2% received ICI in combination with chemotherapy, and 50.6% had received previous radiation therapy: 24.4% with curative intent, and 75.6% with a palliative approach, targeting metastatic lesions.

#### 3.1.2 Twelve-month timepoint analysis cohort

In the 12-month analysis of 246 patients with stage IV disease, 183 patients were excluded because they experienced POD prior to 12 months, resulting in 63 total patients: 23 patients without POD discontinued ICI prior to 12 months and were compared to 40 patients that continued ICI beyond 12 months. The 63 patients had a median age of 68 (range, 44 - 99). 90.5% were white, 50.8% were male, and 81.5% had a history of tobacco use. Adenocarcinoma (79.4%) was the most common histological type of NSCLC, followed by squamous cell carcinoma (9.5%). The most common site of metastasis was bone (24.2%), followed by CNS (15.9%), lymph nodes (14.6%), pleura (including pleural effusion, 13.4%), and contralateral lung (11.0%). 80.9% of patients had stage IV disease at initial diagnosis. 66.7% of patients had received ICI as the first-line systemic therapy, and 25.4% received it as their second. Pembrolizumab was the most frequently used ICI (82.4%), followed by atezolizumab (11.8%) and nivolumab (4.9%). 41.3% used ICI in combination with chemotherapy, and 44.4% had received previous radiation therapy, 28.6% with curative intent and 71.4% with a palliative approach that targeted metastatic lesions (**Table 1**).

**Table 1:** Characteristics of patients with no POD who discontinued vs continued immune checkpoint inhibitor therapy.

6 Month timepoint	Discontinued ICI (≤ 6M of ICI; n=17, unless specified)	Continued ICI (> 6M of ICI; n=64, unless specified)	Overall (n=81, unless specified)
Median age - yr (Range) Male sex – no. (%) Race/ethnicity – no. (%) White Black Hispanic Asian/Pacific Islander Other Cancer Type – no. (%) Adenocarcinoma Squamous Cell Carcinoma Sarcomatoid Other Tobacco use – no. (%) Former Current Never Site of metastasis at initial diagnosis– no. (%) Bone (including spine) CNS Lymph node Contralateral lung Pleura (including pleural effusion) Adrenal gland Liver Other ICI – no. (%) Pembrolizumab Atezolizumab Nivolumab Initial diagnosis stage IV Combination with chemotherapy Previous radiation therapy Curative intent Palliative intent Number of previous systemic therapies 0 1 2 ECOG at ICI initiation 0 1 2 PD-L1% 0 1–49 ≥ 50 unreported	66 (47–88) 8 (47.1) 16 (94.1) 0 0 1 (5.9) 0 13 (76.5) 3 (17.6) 0 1 (5.9) 13 (76.5) 1 (5.9) 3 (17.6) *(n=23)* 8 (34.8) 2 (8.7) 3 (13.0) 3 (13.0) 3 (13.0) 1 (4.3) 2 (11.8) 1 (4.3) *(n=18)* 15 (83.3) 2 (11.1) 1 (5.6) 13 (76.5) 9 (50) 4 (22.2) 1 (25) 3 (75) 13 (76.5) 4 (23.5) 0 9 (52.9) 6 (35.3) 2 (11.8) 5 (29.4) 3 (17.6) 5 (29.4) 4 (23.5)	67 (46–94) 35 (54.6) 53 (82.8) 4 (6.3) 2 (3.1) 1 (1.6) 4 (6.3) 52 (81.3) 6 (9.4) 2 (3.1) 4 (6.3) 47 (73.4) 5 (7.8) 12 (18.8) *(n=72)* 15 (20.8) 14 (19.4) 12 (16.7) 9 (12.5) 9 (12.5) 5 (6.9) 3 (4.2) 5 (6.9) *(n=69)* 56 (81.2) 9 (13.0) 4 (5.8) 46 (71.9) 26 (40.6) 37 (57.8) 11 (29.7) 26 (70.3) 36 (56.3) 20 (31.3) 8 (12.5) 40 (62.5) 21 (32.8) 3 (4.7) 13 (20.3) 18 (28.1) 20 (31.3) 13 (20.3)	67 (46–94) 43 (53.1) 69 (85.2) 4 (4.9) 2 (2.5) 2 (2.5) 4 (4.9) 65 (80.2) 9 (11.1) 2 (2.5) 5 (6.2) 60 (74.1) 6 (7.4) 15 (18.5) *(n=95)* 23 (24.2) 16 (16.8) 15 (15.8) 12 (12.6) 12 (12.6) 6 (7.4) 5 (6.2) 6 (7.4) (n=87) 71 (81.6) 11 (12.6) 5 (5.7) 61 (75.3) 35 (43.2) 41 (50.6) 10 (24.4) 31 (75.6) 49 (60.5) 24 (29.6) 8 (9.9) 49 (60.5) 27 (33.3) 5 (6.2) 18 (22.2) 21 (25.9) 25 (30.9) 17 (21.0)
12 Month timepoint	Discontinued ICI (≤ 12M of ICI; n=23, unless specified)	Continued ICI (> 12M of ICI; n=40, unless specified)	Overall (n=63, unless specified)
Median age - yr (Range) Male sex – no. (%) Race/ethnicity – no. (%) White Black Hispanic Asian/Pacific Islander Other Cancer Type – no. (%) Adenocarcinoma Squamous Cell Carcinoma Sarcomatoid Other Tobacco use – no. (%) Former Current Never unknown Site of metastasis at initial diagnosis– no. (%) Bone (including spine) CNS Lymph node Contralateral lung Pleura (including pleural effusion) Adrenal gland Liver Other ICI – no. (%) Pembrolizumab Atezolizumab Nivolumab Initial diagnosis stage IV Combination with chemotherapy Previous radiation therapy Curative intent Palliative intent Number of previous systemic therapies 0 1 2 ECOG at ICI initiation 0 1 2 PD-L1% 0 1–49 ≥ 50 unreported	72 (45–99) 12 (52.2) 22 (95.7) 0 0 1 (4.3) 0 18 (78.3) 3 (13.0) 0 2 (8.7) 17 (73.9) 1 (4.3) 4 (17.4) 1 (4.3) *(n=28)* 12 (42.9) 2 (7.1) 4 (14.3) 3 (10.7) 3 (10.7) 1 (3.6) 2 (7.1) 1 (3.6) *(n=24)* 20 (83.3) 3 (12.5) 1 (4.2) 19 (82.6) 10 (43.5) 10 (43.5) 3 (30) 7 (70) 18 (78.3) 4 (17.4) 1 (4.3) 11 (47.8) 9 (39.1) 3 (13.0) 5 (21.7) 5 (21.7) 8 (34.8) 5 (21.7)	66 (44–87) 20 (50) 35 (87.5) 3 (7.5) 2 (5) 0 0 32 (80) 3 (7.5) 2 (5) 3 (7.5) 29 (72.5) 3 (7.5) 8 (20) 0 *(n=54)* 8 (14.8) 11 (27.5) 8 (20) 6 (15) 8 (20) 2 (5) 6 (15) 5 (12.5) *(n=44)* 36 (81.8) 5 (11.4) 3 (6.8) 32 (80) 16 (40) 18 (45) 5 (27.8) 13 (72.2) 24 (60) 12 (30) 4 (10) 26 (65) 12 (30) 2 (5) 7 (17.5) 10 (25) 16 (40) 7 (17.5)	68 (44–99) 32 (50.8) 57 (90.5) 3 (4.8) 2 (3.2) 1 (1.6) 0 50 (79.4) 6 (9.5) 2 (3.2) 5 (7.9) 46 (73.0) 4 (6.3) 12 (19.0) 1 (1.6) *(n=82)* 20 (24.4) 13 (15.9) 12 (14.6) 9 (11.0) 11 (13.4) 3 (3.7) 8 (9.8) 6 (7.3) *(n=68)* 56 (82.4) 8 (11.8) 4 (5.9) 51 (80.9) 26 (41.3) 28 (44.4) 8 (28.6) 20 (71.4) 42 (66.7) 16 (25.4) 5 (7.9) 37 (58.7) 21 (33.3) 5 (7.9) 12 (19.0) 15 (23.8) 24 (38.1) 12 (19.0)

M, month; ICI, immune checkpoint inhibitor; CNS, central nervous system; ECOG, Eastern Cooperative Oncology Group Score; PD-L1, programmed death-ligand 1.

#### 3.1.3 Performance status and PD-L1 expression

The ECOG score at ICI initiation was recorded for each patient. ECOG scores of 1–2 (no patients started ICI with ECOG scores greater than 2) were not statistically different when comparing the ICI discontinuation with the ICI continuation group at both 6 months (47.1% vs 37.5%, p=.474) and 12 months (52.2% vs 40.0%, p=0.183). Furthermore, worst reported ECOG score during a patient’s treatment journey was recorded for each patient. ECOG scores of 2–4 were not statistically different when comparing the ICI discontinuation with ICI continuation group at both 6 months (52.9% vs 45.7%, p=0.496) and 12 months (47.8% vs 45.0%, p=0.920).

There was also no statistical difference in the PD-L1 expression between discontinued and continued ICI groups for both timepoints (**Supplementary Table 1**).

### 3.2 Reasons for immune checkpoint inhibitor therapy discontinuation

The most common reason for discontinuation of ICI other than POD was irAEs, with 9/17 patients (52.9%) discontinuing ICI due to an irAE prior to 6 months and 11/23 patients (47.8%) prior to 12 months. Other reasons for discontinuing ICI were non-immune-related AEs with 4/17 patients (23.5%) in the 6-month analysis and 5/23 patients (21.7%) in the 12-month analysis. The non-immune-related AEs included renal failure (determined to be from chemotherapy), infections, non-inflammatory chest pain, or fatigue. 1/17 patients (5.9%) discontinued ICI due to stable disease in the 6-month analysis and 4/23 patients (17.4%) in the 12-month analysis. In both analyses, 1 patient discontinued ICI due to identification of a targetable mutation and 1 patient discontinued ICI due to clinical deterioration despite no disease progression (**Table 2**).

**Table 2:** Reasons for discontinuing immune checkpoint inhibitor therapy other than progression of disease.

Reason	≤ 6M of ICI (n=17)	≤ 12M of ICI (n=23)
Immune related adverse event	9	11
Non-immune related adverse events	4	5
Stable disease	1	4
Targetable mutation identified	1	1
Deterioration	2	2

M, month; ICI, immune checkpoint inhibitor therapy.

### 3.3 Progression free survival and overall survival

**Figure 2:**
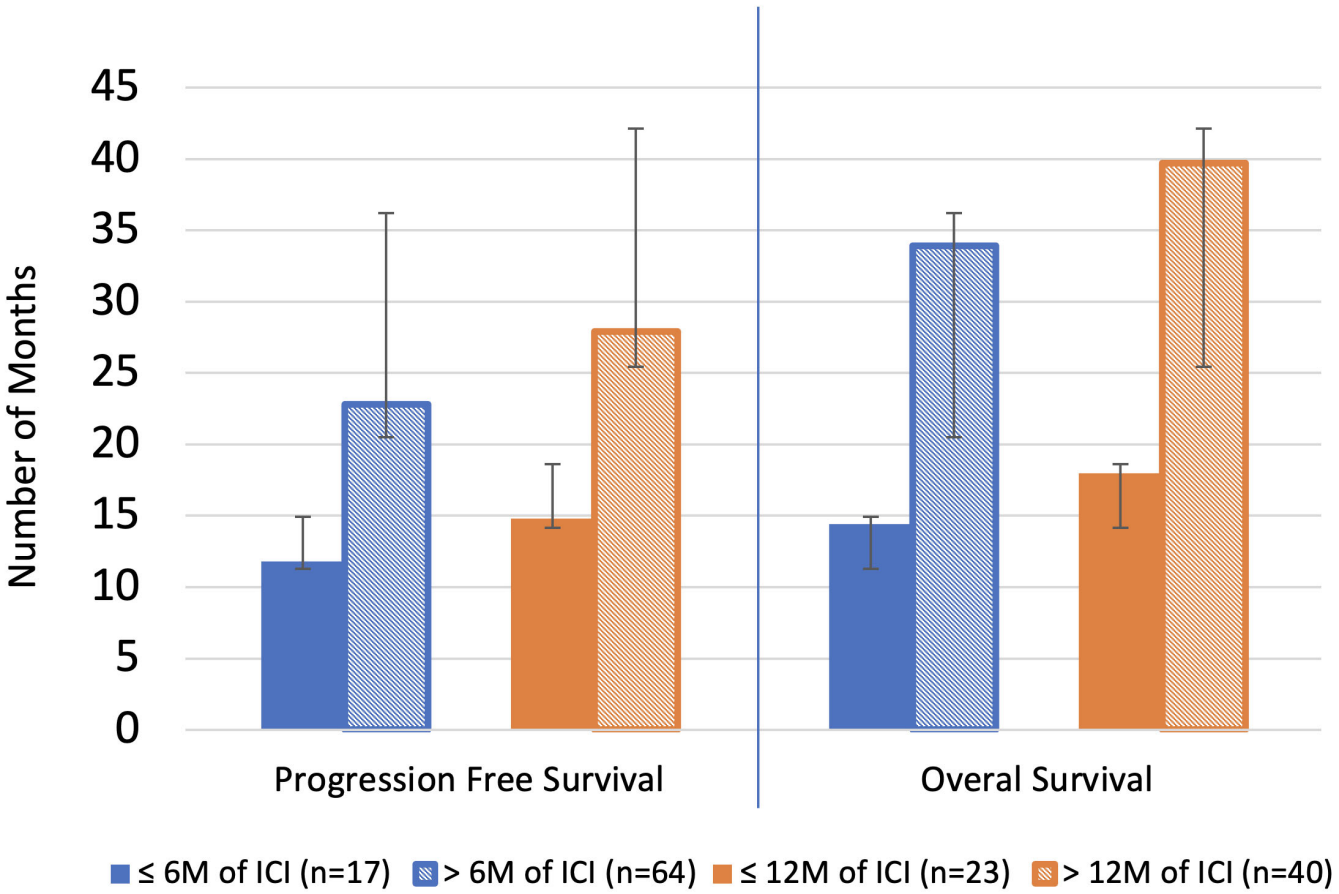
Overall survival and progression free survival. Blue line separates individual analyses. M, month; ICI, immune checkpoint inhibitor therapy.

Patients who continued treatment had a longer duration of PFS compared to those who discontinued treatment at 6 months (22.8 months vs 11.8 months, p=1.1E-04) and 12 months (27.9 months vs 14.8 months, p=1.1E-04). Patients who continued treatment had a longer duration of OS compared to those who discontinued treatment at 6 months (33.9 months vs 14.4 months, p= 7.2E-08) and 12 months (39.7 months vs 18.0 months, p= 2.0E-07). The average duration of ICI was 20.9 months vs 3.2 months in the continued and discontinued groups, respectively, at 6 months and 26.1 months vs 4.9 months, respectively, at 12 months. (**Table 3**, **Figure 2**).

**Table 3:** Outcomes of patients with no progression of disease by immune checkpoint inhibitor therapy duration.

Timepoint	Groups	n	Months on ICI	Months to POD	Months to Death	Last Known Alive	Overall Survival
6 Month	≤ 6M of ICI	17	3.2	11.8	9.9	17.5	14.4
	> 6M of ICI	64	20.9	22.8	29.9	37.0	33.9
	p-value		4.9 E-15	0.001	2.4 E-06	1.7 E-04	7.2 E-08
12 Month	≤ 12M of ICI	23	4.9	14.8	18.6	17.4	18.0
	> 12M of ICI	40	26.1	27.9	35.5	42.0	39.7
	p-value		3.3 E-12	5.8 E-05	0.003	1.5 E-06	2.0 E-07

M, month; ICI, immune checkpoint inhibitor therapy; POD, progression of disease.

A sub-analysis was performed for the 6-month timepoint by restricting the continued ICI group to receiving less than 12 months of total ICI. This was performed to evaluate if there is a survival benefit to continuing ICI for only a limited number of months beyond 6 months. Patients who received > 6-months but ≤ 12 months of ICI trended toward longer PFS compared to patients who discontinued ICI prior to 6 months (16.7 months vs 11.8 months, p=0.234) and experienced a significant longer OS (24.6 months vs 14.04 months, p= 0.013). The average duration of ICI was 10.4 months vs 3.2 months in the continued and discontinued groups, respectively (**Supplementary Tables 2**-**4**).

### 3.4 Immune-related adverse events

**Figure 3:**
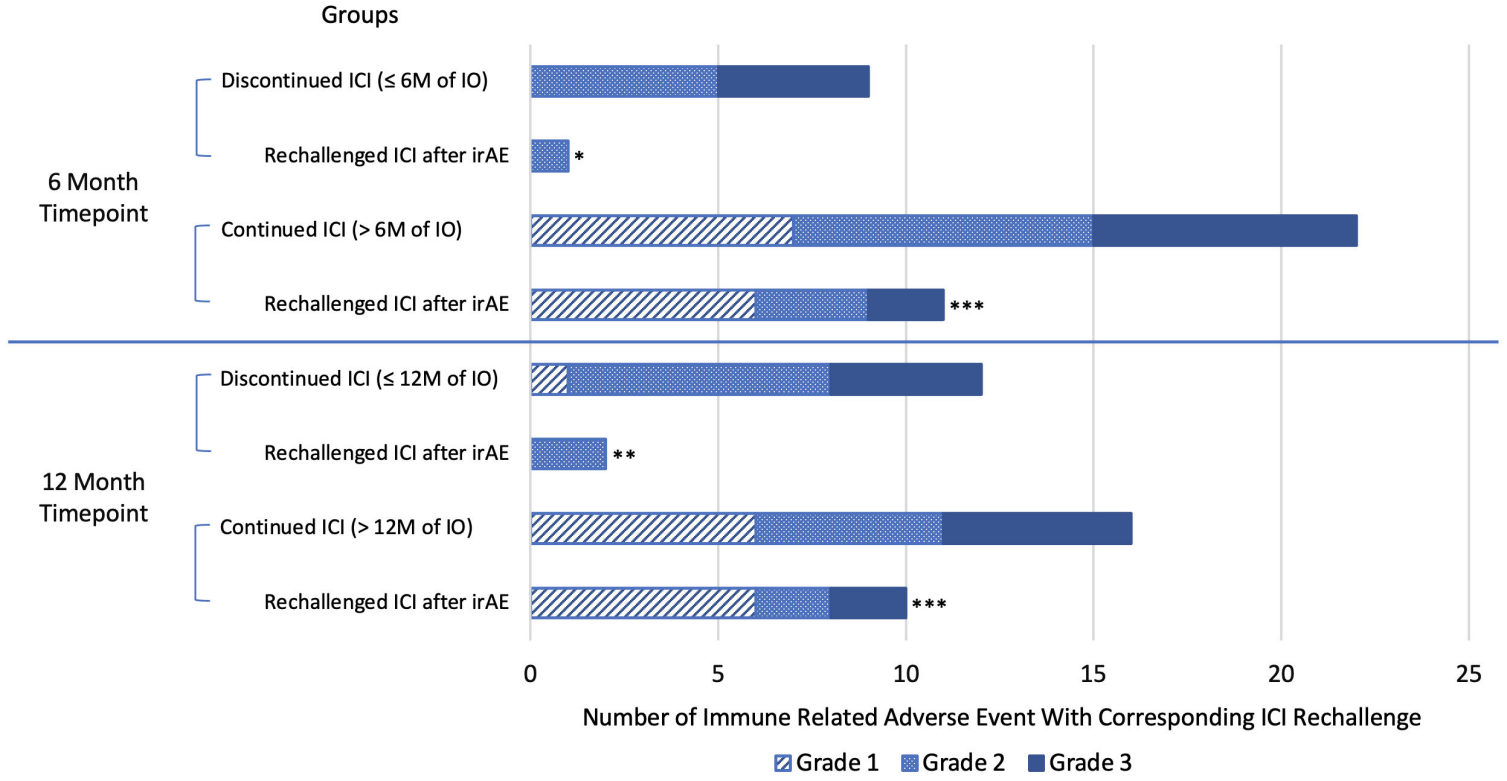
Number of immune-related adverse events with corresponding number of patients that rechallenged/continued immune checkpoint inhibitor therapy by grade. Blue line separates individual analyses. *Rechallenged/continued ICI after irAE, but stopped at or before 6 months. ** Rechallenged/continued ICI after irAE, but stopped at or before 12 months. *** Statistically significant greater proportion of patients continued ICI after experiencing an irAE in the ICI continuation groups compared to the ICI discontinuation group. irAE, immune-related adverse event; M, month; ICI, immune checkpoint inhibitor therapy.

Immune-related AEs of any grade occurred in 52.9% of patients who discontinued ICI vs 34.4% (p=0.136) of patients who continued ICI at 6 months. Similarly, irAE of any grade occurred in 52.2% of those who discontinued ICI vs 37.5% (p=0.257) of those who continued ICI at 12 months. In the 6-month analysis, the proportion of patients that experienced ≥ grade 2 irAEs was significantly greater in the group that discontinued ICI vs those that continued: 9/17 (52.9%) vs 15/64 (23.4%), p = 0.018. Of the patients that experienced a grade 1–3 irAE, a significantly higher number of patients continued or rechallenged treatment after experiencing an irAE in the groups that continued ICI versus those that discontinued ICI at 6 months (11/22, 50% vs 1/9, 11.1%; p-value = 0.044) and 12 months (10/15, 66.7% vs 2/12, 17.7%; p= 0.009). At both the 6- and 12-month analyses, the 2 patients with grade 3 mucositis or hepatitis that rechallenged ICI did not experience another irAE. Also, in both analyses, 4/12 (33.3%) patients that continued ICI after an irAE paused ICI for 1–3 months prior to restarting ICI, and 2/12 (16.7%) patients experienced another irAE, the second being a grade 1 or 2 irAE (**Table 4**, **Figure 3**).

**Table 4:** Immune-related adverse event by grade and proportion that continued immune checkpoint inhibitor therapy after adverse event.

Time-point	Group	Grade 1			Grade 2			Grade 3			Grade ≥ 2		All Grades Together	
		irAE type	n, %	Patients that cont ICI after irAE^1^; n, %	irAE type	n, %	Patients that cont ICI after irAE^1^; n, %	irAE type	n, %	Patients that cont ICI after irAE^1^; n, %	n, %	Patients that cont ICI after irAE^1^; n, %	n, %	Patients that cont ICI after irAE^1^; n, %
6 M	Discontinued ICI (≤ 6M of ICI)				Renal insufficiency, colitis, thyroiditis^2^, hepatitis, pneumonitis	5/17, 29.4%	1/5, 20%	Arthritis/ myositis, pneumonitis, colitis, hepatitis	4/17, 25.3%	0/4, 0%	9/17, 52.9%	1/9, 11.1%	9/17, 52.9%	1/9, 11.1%
	Continued ICI (> 6M of ICI)	AKI, colitis, colitis cutaneous reaction X2, pneumonitis, hepatitis	7/64, 10.9%	6/7, 85.7%	Colitis x3, nephritis, thyroiditis^3^, thyroiditis, adrenal insufficiency, pneumonitis	8/64, 12.5%	3/8, 37.5%	Hepatitis x2, adrenal insufficiency, pneumonitis x3, mucositis	7/64, 10.9%	2/7, 28.6%	15/64, 23.4%	5/15, 33.3%	22/64, 34.4%	11/22, 50%
	p-value					0.091	0.506		0.126		0.018	0.224	0.136	0.044
12 M	Discontinued ICI (≤ 12M of ICI)	AKI	1/23, 4.3%	0/1, 0%	Renal insufficiency, colitis x2, thyroiditis^2^, hepatitis, pneumonitis, pneumonitis/adrenal insufficiency	7/23, 25%	2/7, 28.6%	Colitis, arthritis/ myositis, pneumonitis, hepatitis	4/23, 17.4%	0/4, 0%	11/23, 47.8%	2/11, 18.2%	12/23, 52.2%	2/12, 17.7%
	Continued ICI (> 12M of ICI)	Colitis X2, cutaneous reaction x2, pneumonitis, hepatitis	6/40, 15%	6/6, 100%	Colitis, nephritis, thyroiditis, thyroiditis^3^, pneumonitis	5/40, 12.5%	2/5, 40%	Hepatitis x2, adrenal insufficiency, mucositis, pneumonitis	5/40, 12.5%	2/5, 40%	10/40, 25%	4/10, 40%	15/40, 37.5%	10/15, 66.7%
	p-value		0.195			0.183	0.679		0.593		0.064	0.269	0.257	0.009

1 Continued ICI after experiencing an irAE with or without a pause in treatment.

2 Experienced grade 2 thyroiditis and subsequently experienced grade 2 and then grade 3 colitis.

3 Experienced grade 2 thyroiditis and subsequently experienced grade 1 colitis and then grade 1 arthritis.

M, month; ICI, immune checkpoint inhibitor therapy; irAE, immune related adverse event; cont, continue; AKI, acute kidney injury.

## 4 Discussion

Suspension of ICI for reasons other than POD, such as treatment-related adverse events, is a commonly encountered clinical scenario for stage IV mNSCLC patients. This real-world analysis aimed to determine whether there is a benefit in PFS and OS in patients who can continue ICI versus those who discontinue ICI prior to either 6 or 12 months. After excluding patients with POD prior to 6 or 12 months, the patients who continued ICI beyond 6 months and 12 months were found to have significant increases in PFS, averaging 11 and 13.1 more months, respectively, compared to the groups that discontinued ICI. Similarly, OS was significantly increased in the groups that continued ICI beyond 6 or 12 months, with 19.5-months-longer OS in the 6-month cohort and 21.7-months-longer OS in the 12-month cohort. There was also shown to be a significant difference in OS in the sub-analysis of the 6-month cohort when considering the group that continued ICI > 6 months but < 12 months, averaging 10.2-months-longer OS. Therefore, there appears to be a survival benefit of continuing ICI even for a limited duration past 6 months. The observed survival benefit cannot be explained by differences in patient characteristics between groups, including no significant differences in performance status, PD-L1 expression, or irAE incidence. Therefore, these results are relevant to patients who have no POD and are inquiring if they should continue therapy. Patients who continued ICI were found to have clinically meaningful prolonged PFS and increased OS.

After excluding patients with POD, patients were found to discontinue ICI most commonly due to irAEs. There were no statistically significant differences in the incidence of irAEs between groups that stopped or continued ICI at the 6- or 12-month timepoints according to individual grade or total irAEs; however, the frequency of grade ≥ 2 irAEs was significantly higher in those that discontinued ICI prior to 6 months. In the 6-month analysis with restriction of ICI to less than 12 months, there were no grade 3 irAEs, compared to 5 irAEs that occurred in 6 months or fewer; however, there were 5 grade 3 irAEs that occurred after 12 months. This pattern suggests that the grade 3 irAEs occurred in a bimodal distribution with peaks in the first 6 months and after 12 months of ICI. Although grade 3 irAEs were observed again after 12 months, the majority of grade 1 irAEs also occurred after 12 months. Experiencing an early grade 3 irAE may have contributed to patients discontinuing ICI early and experiencing decreased PFS and OS.

More patients continued or rechallenged with ICI after experiencing irAEs in the groups that continued ICI in both the 6- and 12-month analyses, but more patients experienced grade 1 irAEs in the ICI continuation groups. When grade 1 irAEs were excluded, there were no statistically significant differences in ICI continuation or rechallenge between groups, although results trended toward higher rates of ICI continuation or rechallenge in the groups that continued ICI beyond 6 and 12 months. Therefore, we conclude that continuing or rechallenging ICI after irAEs contributed to longer ICI use, resulting in better survival in the groups that continued ICI past 6 and 12 months.

In a combination of 4 independent studies, 351 patients were rechallenged with ICI after experiencing ≥ a grade 2 irAE and found that 39–78% of patients experienced another irAE, but concluded that the risk-reward of rechallenge was acceptable (13–16). The recurrent or new irAEs that patients experienced were reported as not as severe as the first (14) and manageable with 84% of the irAEs reported as resolved or improved to grade 1 (16). Another study identified over 24,000 cases of irAEs and found that a rechallenge of ICI after irAEs resulted in 28.8% of patients experiencing the same irAE that required discontinuation of ICI, concluding that rechallenge could be considered (17). Guidelines for management of grade 3 irAE generally advise definite ICI discontinuation (18). However, the evaluation of this topic remains ongoing. ICI rechallenge was stated to be relatively safe after grade 2 and 3 irAEs except for cardiac and neurological irAEs (13). A review article on rechallenging ICI following severe irAE concluded that ICI rechallenge after temporary discontinuation of grade 3–4 irAE is feasible in most patients (19). In our study, the 2 patients who experienced a grade 3 irAE of mucositis or hepatitis continued therapy after 1–3 months and did not experience another irAE. We found favorable results among the 12 patients that continued/or rechallenged ICI after an irAE with only 2 patients (16.7%) in both the 6- and 12-month cohorts experiencing a subsequent grade 1 or 2 irAE. The outcomes of our study show increased PFS and OS in patients that continue ICI past 6 and 12 months; therefore, continuation or rechallenge of ICI should be considered.

Our study differs from other studies that analyze survival post-irAEs. Many studies compare survival outcomes between groups of patients that experience irAEs to those who do not, reporting that some patients experience a durable immune response after discontinuing ICI that is associated with their irAEs (20–22). In our study, there is no statistical difference in the total incidence of irAEs between the ICI continued and discontinued groups. Thus, the incidence of irAEs did not contribute to increased survival in the ICI continued groups. However, there was a significant difference in the percentage of patients that continued/rechallenged ICI after an irAE, suggesting that continued/rechallenged ICI after experiencing an irAE offers some survival benefit. Furthermore, patients that experience no response to ICI prior to irAEs may prove to respond better to ICI after rechallenge (16). The authors encourage patients and oncology providers to work together to overcome irAEs and continue ICI, if deemed appropriate, with or without a pause in therapy, in the event of grade 1 and 2 irAEs. Oncology providers should also discuss rechallenging ICI after experiencing a grade 3 irAE, taking into consideration the type of irAE. Pneumonitis is one of the irAEs that poses a higher risk for rechallenge (13). Checkpoint inhibitor-induced colitis has been reported as having higher recurrence rates after rechallenge (15, 17).

Other reasons for discontinuing ICI are non-irAEs. This group included patients that discontinued ICI due to symptoms that were not specific to an irAE, such as fatigue, or adverse events that were determined to be due to chemotherapy, such as biopsy-proven acute kidney injury. These patients should consider continuing ICI. Another common reason for discontinuing ICI that was not observed in this cohort is the drug cost, affecting its use in some countries more than others due to differences in insurance coverage and access (23, 24). These are challenges that patient care teams should help navigate with the goal to continue ICI.

Patients that stop ICI prior to 6 months and 12 months due to stable disease may also consider continuing therapy. More patients stopped ICI due to stable disease in the 12-month group compared to the 6-month group. The optimal duration of ICI is still debated. NCCN guidelines suggest that patients with stage IV NSCLC should continue ICI for 24 months (7). Furthermore, it has been shown that continuing ICI past 24 months does not offer significant survival benefits (9, 10). Although 24 months has been suggested by the guideline, assessment of the survival benefit of continuing ICI for varying durations < 24 months has been limited. The study CheckMate 153 investigated survival outcomes by comparing continuous versus fixed 12-months of nivolumab in patients with advanced NSCLC. It found a longer PFS in the continuous treatment group (24.7 months vs. 9.4 months) and longer OS (not reached vs. 32.5 months) (25). This study is unique compared to ours because every patient received at least 12 months of treatment and did not evaluate other types of ICIs. Our study confirms from real-world data that continuing ICI use beyond both 6 and 12 months provides clinically significant, better survival outcomes.

The study is limited by its retrospective design and small sample size, which limited the ability to perform sub-analyses. The study is limited to a single institution, although 3 clinical sites were included. The patient population is predominantly Caucasian, which reduced the generalizability of the results to other races/ethnicities. Genetic profiling was limited to PD-L1 assessment, a more robust molecular profiling may have impacted results and improved understanding of ICI effectiveness. Future directions include a prospective study design to validate and strengthen conclusions. Future studies should include larger sample sizes with a stratified analysis by treatment type and previous systemic therapy use.

## 5 Conclusions

Patients with stage IV NSCLC and no POD who continued checkpoint inhibitor ICI beyond 6 months and 12 months experienced a significant increase in PFS and OS compared to patients who discontinued ICI early, with more significant increases in those who continued ICI past 12 months. This information is clinically significant for patients with irAEs, non-irAEs, or stable disease prior to 24 months of ICI who are considering early discontinuation of ICI. The shared decision-making process should include a discussion of the survival benefit of continuing ICI past 12 months and offer support to overcome obstacles to treatment, including management of irAEs, particularly grade 1–2 irAEs.

## Data availability statement

The raw data supporting the conclusions of this article will be made available by the authors, without undue reservation.

## Ethics statement

The studies involving humans were approved by Mayo Clinic Institutional Review Board. The studies were conducted in accordance with the local legislation and institutional requirements. Written informed consent for participation was not required from the participants or the participants’ legal guardians/next of kin in accordance with the national legislation and institutional requirements.

## Author contributions

BM: Conceptualization, Data curation, Formal analysis, Investigation, Methodology, Project administration, Visualization, Writing – original draft. TP: Investigation, Writing – review & editing. EW: Investigation, Writing – review & editing. SL: Investigation, Writing – review & editing. GC: Investigation, Writing – review & editing. YZ: Writing – review & editing. RM: Investigation, Validation, Writing – review & editing. YL: Conceptualization, Investigation, Methodology, Supervision, Writing – review & editing.

## References

[1] Sung H Ferlay J Siegel RL Laversanne M Soerjomataram I Jemal A. Global cancer statistics 2020: GLOBOCAN estimates of incidence and mortality worldwide for 36 cancers in 185 countries. CA Cancer J Clin. (2021) 71:209–49. doi: 10.3322/caac.21660 33538338

[2] Novello S Barlesi F Califano R Cufer T Ekman S Levra MG. Metastatic non-small-cell lung cancer: ESMO Clinical Practice Guidelines for diagnosis, treatment and follow-up †. Ann Oncol. (2016) 27:v1–27. doi: 10.1093/annonc/mdw326 27664245

[3] Hanna NH Schneider BJ Temin S Baker S Brahmer J Ellis PM. Therapy for stage IV non-small-cell lung cancer without driver alterations: ASCO and OH (CCO) joint guideline update. J Clin Oncol. (2020) 38:1608–32. doi: 10.1200/JCO.19.03022 31990617

[4] Research C for DE and. Pembrolizumab (KEYTRUDA) Checkpoint Inhibitor. FDA [Internet] (2019). Available online at: https://www.fda.gov/drugs/resources-information-approved-drugs/pembrolizumab-keytruda-checkpoint-inhibitor.

[5] Post TA. FDA Expands Pembrolizumab Indication for NSCLC in First-Line Setting - The ASCO Post [Internet] . Available online at: https://ascopost.com/issues/may-25–2019/fda-expands-pembrolizumab-indication-for-nsclc-in-first-line-setting/.

[6] Ferrara R Imbimbo M Malouf R Paget-Bailly S Calais F Marchal C. Single or combined immune checkpoint inhibitors compared to first-line platinum-based chemotherapy with or without bevacizumab for people with advanced non-small cell lung cancer. Cochrane Database Syst Rev. (2020) 2020:CD013257. doi: 10.1002/14651858.CD013257.pub2 PMC8094159 33316104

[7] Ettinger DS Wood DE Riely. GJ. NCCN clinical practice guidelines in oncology (NCCN guidelines®) version 2.2024 non-small cell lung cancer. SYSTEMIC THERAPY FOR ADVANCED OR METASTATIC DISEASE – MAINTENANCE. [Internet]. Natl Compr Cancer Network. (2024).

[8] Putzu C Canova S Paliogiannis P Lobrano R Sala L Cortinovis DL. Duration of immunotherapy in non-small cell lung cancer survivors: A lifelong commitment? Cancers. (2023) 15:689. doi: 10.3390/cancers15030689 36765647 PMC9913378

[9] Sun L Bleiberg BA Hwang WT Marmarelis ME Langer CJ Singh AP. Association between duration of immunotherapy and overall survival in advanced non–small-cell lung cancer. JCO. (2023) 41:9101–1. doi: 10.1200/JCO.2023.41.16_suppl.9101 PMC10240399 37270700

[10] Ardin C Humez S Leroy V Ampere A Bordier S Escande F. Pursuit or discontinuation of anti-PD1 after 2 years of treatment in long-term responder patients with non-small cell lung cancer. Ther Adv Med Oncol. (2023) 15:17588359231195600. doi: 10.1177/17588359231195600 37720494 PMC10501064

[11] Antonia SJ Villegas A Daniel D Vicente D Murakami S Hui R. Durvalumab after chemoradiotherapy in stage III non–small-cell lung cancer. New Engl J Med. (2017) 377:1919–29. doi: 10.1056/NEJMoa1709937 28885881

[12] Spigel DR Faivre-Finn C Gray JE Vicente D Planchard D Paz-Ares L. Five-year survival outcomes from the PACIFIC trial: durvalumab after chemoradiotherapy in stage III non–small-cell lung cancer. JCO. (2022) 40:1301–11. doi: 10.1200/JCO.21.01308 PMC9015199 35108059

[13] Kartolo A Holstead R Khalid S Emack J Hopman W Baetz T. Safety of immunotherapy rechallenge after immune-related adverse events in patients with advanced cancer. J Immunother. (2021) 44:41–8. doi: 10.1097/CJI.0000000000000337 32815895

[14] Simonaggio A Michot JM Voisin AL Le Pavec J Collins M Lallart A. Evaluation of readministration of immune checkpoint inhibitors after immune-related adverse events in patients with cancer. JAMA Oncol. (2019) 5:1310–7. doi: 10.1001/jamaoncol.2019.1022 PMC6555478 31169866

[15] Allouchery M Lombard T Martin M Rouby F Sassier M Bertin C. Original research: Safety of immune checkpoint inhibitor rechallenge after discontinuation for grade ≥2 immune-related adverse events in patients with cancer. J Immunother Cancer. (2020) 8(2):e001622. doi: 10.1136/jitc-2020-001622 33428586 PMC7768965

[16] Santini FC Rizvi H Plodkowski AJ Ni A Lacouture ME Gambarin-Gelwan M. Safety and efficacy of re-treating with immunotherapy after immune-related adverse events in patients with NSCLC. Cancer Immunol Res. (2018) 6:1093–9. doi: 10.1158/2326-6066.CIR-17-0755 PMC6125223 29991499

[17] Dolladille C Ederhy S Sassier M Cautela J Thuny F Cohen AA. Immune checkpoint inhibitor rechallenge after immune-related adverse events in patients with cancer. JAMA Oncol. (2020) 6:865–71. 10.1001/jamaoncol.2020.0726 PMC7163782 32297899

[18] Verheijden RJ van Eijs MJM May AM van Wijk F Suijkerbuijk KPM. Immunosuppression for immune-related adverse events during checkpoint inhibition: an intricate balance. NPJ Precis Onc. (2023) 7:1–11. doi: 10.1038/s41698-023-00380-1 PMC10182067 37173424

[19] Haanen J Ernstoff M Wang Y Menzies A Puzanov I Grivas P. Rechallenge patients with immune checkpoint inhibitors following severe immune-related adverse events: review of the literature and suggested prophylactic strategy. J Immunother Cancer. (2020) 8:e000604. doi: 10.1136/jitc-2020-000604 32532839 PMC7295425

[20] Morimoto K Yamada T Takumi C Ogura Y Takeda T Onoi K. Immune-related adverse events are associated with clinical benefit in patients with non-small-cell lung cancer treated with immunotherapy plus chemotherapy: A retrospective study. Front Oncol. (2021) 11. doi: 10.3389/fonc.2021.630136 PMC8021904 33833990

[21] Toi Y Sugawara S Kawashima Y Aiba T Kawana S Saito R. Association of immune-related adverse events with clinical benefit in patients with advanced non-small-cell lung cancer treated with nivolumab. Oncol. (2018) 23:1358–65. doi: 10.1634/theoncologist.2017-0384 PMC6291330 29934411

[22] Sato K Akamatsu H Murakami E Sasaki S Kanai K Hayata A. Correlation between immune-related adverse events and efficacy in non-small cell lung cancer treated with nivolumab. Lung Cancer. (2018) 115:71–4. doi: 10.1016/j.lungcan.2017.11.019 29290265

[23] Schaft N Dörrie J Schuler G Schuler-Thurner B Sallam H Klein S. The future of affordable cancer immunotherapy. Front Immunol. (2023) 14. doi: 10.3389/fimmu.2023.1248867 PMC10509759 37736099

[24] Yilmaz M Guven Mese S. Durable response after discontinuation of nivolumab therapy in the absence of disease progression or toxicity with two advanced NSCLC patients. J Oncol Pharm Pract. (2020) 26:761–7. doi: 10.1177/1078155219867131 31423946

[25] Waterhouse DM Garon EB Chandler J McCleod M Hussein M Jotte R. Continuous versus 1-year fixed-duration nivolumab in previously treated advanced non–small-cell lung cancer: checkmate 153. JCO. (2020) 38:3863–73. doi: 10.1200/JCO.20.00131 PMC7676888 32910710

