# Supplementary material for: Early discontinuation of immune checkpoint inhibitor therapy prior to disease progression in patients with metastatic non-small cell lung cancer: a survival analysis
Source: Front Oncol. 2024 Jun 21;14:1417175. doi: 10.3389/fonc.2024.1417175 (PMC11224446; doi:10.3389/fonc.2024.1417175)
Supplement: Supplementary file 1 [file Table_1.docx]

Supplemental Table 1: PD-L1 expression comparisons

| Analysis | Pd-L1 expression (%) | Discontinued ICI | Continued ICI | p-value |
| --- | --- | --- | --- | --- |
| 1 |  | ≤6M (n=17) | > 6M (n=64) |  |
|  | 0 | 5 | 13 | .422465 |
|  | 1-49 | 3 | 18 | .380873 |
|  | ≥ 50 | 5 | 20 | .884043 |
| 2 |  | ≤6 M (n=17) | > 6M, <12M  (n=22) |  |
|  | 0 | 5 | 5 | .422465 |
|  | 1-49 | 3 | 7 | .314889 |
|  | ≥ 50 | 5 | 4 | .409145 |
| 3 |  | ≤12M (n=23) | > 12M (n=40) |  |
|  | 0 | 5 | 7 | .679944 |
|  | 1-49 | 5 | 10 | .76985 |
|  | ≥ 50 | 8 | 16 | .446226 |

Abbreviations: M = month, ICI: immune checkpoint inhibitor therapy

Supplemental Table 2: Outcomes of patients with no progression of disease by immune checkpoint inhibitor therapy duration

| **Timepoint** | **Groups** | **n** | **Months on ICI** | **Months to POD** | **Months to Death** | **Last Known Alive** | **Overall Survival** |
| --- | --- | --- | --- | --- | --- | --- | --- |
| 6 Month With 12 Month ICI Restriction | ≤ 6M of ICI | 17 | 3.2 | 11.8 | 9.9 | 17.5 | 14.4 |
|  | > 6M, ≤ 12M of ICI | 22 | 10.4 | 16.7 | 24.8 | 24.4 | 24.6 |
|  | p-value |  | 1.3 E-14 | 0.234 | 0.001 | 0.367 | 0.013 |

Abbreviations: M = month, ICI: immune checkpoint inhibitor therapy, POD = progression of disease.

Supplemental Table 3: Characteristics of patients with PFS who discontinued ICI ≤ 6 months vs continued ICI > 6 months but < 12 months

| 6 Month Timepoint With 12 Month ICI Restriction | Discontinued ICI (≤ 6M of ICI; n=17, unless specified) | Continued ICI (>6M, <12M of ICI; n=22, unless specified) | Overall  (n=39, unless specified) |
| --- | --- | --- | --- |
| Median age - yr (Range)  Male sex – no. (%)  Race/ethnicity – no. (%)  White  Black  Hispanic  Asian / Pacific Islander  Other  Cancer Type – no. (%)  Adenocarcinoma  Squamous Cell Carcinoma  Sarcomatoid  Other  Tobacco use – no. (%)  Former  Current  Never  Site of metastasis at initial diagnosis – no. (%)  Bone (including spine)  CNS  Lymph node  Contralateral lung  Pleura (including pleural effusion)  Adrenal gland  Liver  Other  Immunotherapy – no. (%)  Pembrolizumab  Atezolizumab  Nivolumab  Initial diagnosis stage IV  Combination with chemotherapy  Previous radiation therapy  Curative intent  Palliative intent  Number of previous systemic therapies  0  1  2  Worst reported ECOG  0  1  2  3  4  Unknown  PD-L1 %  0  1-49  ≥ 50  unreported | 66 (47-88)  8 (47.1)  16 (94.1)  0  0  1 (5.9)  0  13 (76.5)  3 (17.6)  0  1 (5.9)  13 (76.5)  1 (5.9)  3 (17.6)  *(n=23)*  8 (34.8)  2 (8.7)  3 (13.0)  3 (13.0)  3 (13.0)  1 (4.3)  2 (11.8)  1 (4.3)  *(n=18)*  15 (83.3)  2 (11.1)  1 (5.6)  15 (88.2)  9 (50)  4 (22.2)  1 (25)  3 (75)  13 (76.5)  4 (23.5)  0  2 (11.8)  5 (29.4)  3 (17.6)  4 (23.5)  2 (11.8)  1 (5.9)  5 (29.4)  3 (17.6)  5 (29.4)  4 (23.5) | 66 (45-87)  14 (63.6)  18 (81.8)  1 (4.5)  0  1 (4.5)  2 (9.1)  20 (90.9)  1 (4.5)  0  1 (4.5)  15 (68.2)  3 (13.6)  4 (18.2)  *(n=19)*  6 (31.6)  3 (15.8)  3 (15.8)  3 (15.8)  1 (5.3)  3 (15.8)  0  0  *(n=23)*  19 (82.6)  3 (13.0)  1 (4.3)  13 (59.1)  10 (45.4)  17 (77.3)  5 (29.4)  12 (70.6)  12 (54.5)  7 (31.8)  3 (13.6)  1 (4.5)  12 (54.5)  6 (27.3)  2 (9.1)  0  1 (4.5)  5 (22.7)  7 (31.8)  4 (18.2)  6 (27.3) | 67 (45-87)  22 (56.4)  34 (87.2)  1 (2.6)  0  2 (5.1)  2 (5.1)  33 (84.6)  4 (10.3)  0  2 (5.1)  28 (71.8)  4 (10.3)  7 (17.9)  *(n=42)*  14 (35.9)  5 (11.9)  6 (14.3)  6 (14.3)  4 (9.5)  4 (9.5)  2 (4.8)  1 (2.4)  *(n=41)*  34 (82.9)  5 (12.2)  2 (4.9)  28 (71.8)  19 (48.7)  21 (53.8)  6 (28.6)  15 (71.4)  25 (64.1)  11 (28.2)  3 (7.7)  3 (7.7)  17 (43.6)  9 (23.1)  6 (15.4)  2 (5.1)  2 (5.1)  10 (25.6)  10 (25.6)  9 (23.1)  10 (25.6) |

Abbreviations: M = month, ICI: immune checkpoint inhibitor therapy, CNS = central nervous system, ECOG = Eastern Cooperative Oncology Group Score, PD-L1 = programmed death-ligand 1

Supplemental Table 4: Immune-related adverse event by grade and proportion that continued ICI after adverse event but less than 12 months

| Timepoint | Group | Grade 1 | | | Grade 2 | | | Grade 3 | | | Grade ≥ 2 | | All Grades Together | |
| --- | --- | --- | --- | --- | --- | --- | --- | --- | --- | --- | --- | --- | --- | --- |
|  |  | irAE type | n, % | Patients with irAE that cont ICI^1^; n, % | irAE type | n, % | Patients with irAE that cont ICI^1^; n, % | irAE type | n, % | Patients with irAE that cont ICI^1^; n, % | n, % | Patients with irAE that cont ICI^1^; n, % | n, % | Patients with irAE that cont ICI^1^; n, % |
| 6 M  With 12 Month ICI Restriction | Discontinued ICI  (≤ 6M of ICI) |  |  |  | Renal insufficiency,  colitis, thyroiditis^2^,  hepatitis,  pneumonitis | 5/17,  29.4% | 1/5,  20% | Arthritis/myositis,  pneumonitis, colitis,  hepatitis | 4/17,  25.3% | 0/4,  0% | 9/17,  52.9% | 1/9,  11.1% | 9/17,  52.9% | 1/9,  11.1% |
|  | Continued ICI  (>6M, <12M of ICI) | AKI | 1/22,  4.5% | 0/1,  0% | Nephritis, colitis x2, adrenal insufficiency | 4/22,  18.2% | 1/4,  25% |  |  |  | 4/22,  18.2% | 1/4,  25% | 5/22,  22.7% | 1/5,  20% |
|  | p-value |  |  |  |  | 0.409 | 0.857 |  |  |  | 0.022 | 0.521 | 0.051 | 0.649 |

1 Continue with or without a pause in treatment

2 Experienced grade 2 thyroiditis and subsequently experienced grade 2 and then grade 3 colitis

Abbreviations: M = month, ICI: immune checkpoint inhibitor therapy, irAE = immune related adverse event, cont = continue, AKI = acute kidney injury
